# Generation of hiPSC-Derived Functional Dopaminergic Neurons in Alginate-Based 3D Culture

**DOI:** 10.3389/fcell.2021.708389

**Published:** 2021-08-02

**Authors:** Valentina Gilmozzi, Giovanna Gentile, Diana A. Riekschnitz, Michael Von Troyer, Alexandros A. Lavdas, Emanuela Kerschbamer, Christian X. Weichenberger, Marcelo D. Rosato-Siri, Simona Casarosa, Luciano Conti, Peter P. Pramstaller, Andrew A. Hicks, Irene Pichler, Alessandra Zanon

**Affiliations:** ^1^Institute for Biomedicine, Eurac Research, Affiliated Institute of the University of Lübeck, Bolzano, Italy; ^2^Department of Cellular, Computational and Integrative Biology-CIBIO, University of Trento, Trento, Italy; ^3^Department of Neurology, University of Lübeck, Lübeck, Germany

**Keywords:** three-dimensional culture, hiPSCs, Parkinson’s disease, microencapsulation, alginate, biomaterials

## Abstract

Human induced pluripotent stem cells (hiPSCs) represent an unlimited cell source for the generation of patient-specific dopaminergic (DA) neurons, overcoming the hurdle of restricted accessibility to disease-affected tissue for mechanistic studies on Parkinson’s disease (PD). However, the complexity of the human brain is not fully recapitulated by existing monolayer culture methods. Neurons differentiated in a three dimensional (3D) *in vitro* culture system might better mimic the *in vivo* cellular environment for basic mechanistic studies and represent better predictors of drug responses *in vivo*. In this work we established a new *in vitro* cell culture system based on the microencapsulation of hiPSCs in small alginate/fibronectin beads and their differentiation to DA neurons. Optimization of hydrogel matrix concentrations and composition allowed a high viability of embedded hiPSCs. Neural differentiation competence and efficiency of DA neuronal generation were increased in the 3D cultures compared to a conventional 2D culture methodology. Additionally, electrophysiological parameters and metabolic switching profile confirmed increased functionality and an anticipated metabolic resetting of neurons grown in alginate scaffolds with respect to their 2D counterpart neurons. We also report long-term maintenance of neuronal cultures and preservation of the mature functional properties. Furthermore, our findings indicate that our 3D model system can recapitulate mitochondrial superoxide production as an important mitochondrial phenotype observed in neurons derived from PD patients, and that this phenotype might be detectable earlier during neuronal differentiation. Taken together, these results indicate that our alginate-based 3D culture system offers an advantageous strategy for the reliable and rapid derivation of mature and functional DA neurons from hiPSCs.

## Introduction

In biomedical research, key features of human pluripotent stem cells (hPSCs), including human embryonic stem cells (hESCs) and human induced pluripotent stem cells (hiPSCs), are their self-renewal capability and the competence to generate unlimited numbers of specialized cell types. These intrinsic properties enable their use as a unique tool for *in vitro* modeling of a large number of diseases, and high throughput drug screening. This is especially important when affected tissues are not easy to obtain, as in the case of Parkinson’s disease (PD) (Fox et al., 2014; Tabar and Studer, 2014; Playne and Connor, 2017; Blau and Daley, 2019).

PD is the second most frequent neurodegenerative disorder affecting 2–3% of the population over 65 years (Poewe et al., 2017). It is clinically characterized by resting tremor, rigidity, bradykinesia, and postural instability (Hoehn and Yahr, 1967). Pathologically, it is confirmed by loss of dopaminergic (DA) neurons in the *substantia nigra pars compacta* (*SNpc*) and the presence of α-synuclein-positive inclusions in the cytoplasm of neurons, termed Lewy bodies (Spillantini et al., 1998). While about 90% of PD cases are classified as idiopathic, in the past two decades, inherited mutations in more than 20 genes have been linked to rare, familial forms of PD and parkinsonism (Blauwendraat et al., 2020). These genetic findings have led to significant advancement of the understanding of the molecular pathways contributing to the loss of DA neurons. Disease modeling efforts have uncovered that midbrain DA neurons generated from PD patients-derived hiPSCs exhibit mitochondrial dysfunction and α-synuclein aggregation as major cellular disease phenotypes (Devi et al., 2008; Byers et al., 2011; Cooper et al., 2012; Imaizumi et al., 2012; Ryan et al., 2013; Flierl et al., 2014; Shaltouki et al., 2015; Chung et al., 2016; Kouroupi et al., 2017).

Although substantial advances in the two-dimensional (2D) cell culture system for an efficient differentiation of hiPSCs into midbrain DA neurons via a floor-plate intermediate have been achieved (Kriks et al., 2011; Doi et al., 2014; Hallett et al., 2014; Steinbeck et al., 2015; Chen et al., 2016; Kirkeby et al., 2017; Gantner et al., 2020; Song et al., 2020), one of the major remaining challenges is the possibility to produce large amounts of DA neurons with high efficiency and reproducibility. In fact, many of the existing differentiation protocols still show a varying efficiency and yield neurons with variable degrees of maturation. Moreover, in routinely used 2D culture approaches, cells are grown and differentiated on rigid plastic surfaces, where they form a monolayer, which does not fully maintain cell-cell and cell-matrix interactions and which does not fully recapitulate their *in vivo* counterparts in terms of functionality (Edmondson et al., 2014). Consequently, cells grown on 2D surfaces exhibit non-physiological responses affecting gene expression patterns, cell growth, migration, differentiation and survival (Cukierman et al., 2002; Geckil et al., 2010; Knight and Przyborski, 2015). The behavior of cells, particularly stem cells, is impacted by the mechanical properties of the surrounding microenvironment, and the early neurogenic differentiation of hPSCs has been shown to be supported by soft substrates with stiffnesses comparable to neural tissue (Saha et al., 2008; Keung et al., 2012). Therefore, the transition to 3D cell culture approaches is critical for modeling cellular and molecular aspects of the disease with more mature neurons showing higher levels of functionality. Recent studies have reported the *in vitro* self-assembling capacity of differentiating hPSCs to generate midbrain organoids (Jo et al., 2016; Monzel et al., 2017; Smits et al., 2019), recapitulating cell types and structural organization of the developing midbrain tissue. Nevertheless, several limitations, including heterogeneity and limited level of maturity and accessibility, still represent real challenges for this system thus limiting its full exploitation in translational studies, such as drug screening or regenerative medicine approaches. In a complementary approach, different biomaterial scaffolds, such as hydrogels, have been exploited to generate 3D cultures that mimic the physiological microenvironment, thereby improving differentiation of hPSCs to obtain neurons with increased functionality and better suitability for drug and toxicity screenings (Dawson et al., 2008; Prewitz et al., 2012). In this scaffold-assisted differentiation approaches, cells are encapsulated in the hydrogel, where they form several clusters. While organoids are considered to have a high similarity to a “physiological” *in vivo* environment, hydrogel-based 3D differentiations are suited for more specific differentiation approaches. Previous studies have shown that human and mouse ESCs (hESCs, mESCs) as well as hiPSCs can be successfully cultured and differentiated into neural lineages by using alginate-based biomaterials (Li et al., 2011; Addae et al., 2012; Lu et al., 2012; Kim et al., 2013; Bozza et al., 2014). Alginate is a linear polysaccharide extracted from marine brown algae (Pawar and Edgar, 2012) known for its ability to form hydrogels at physiological conditions enabling easy cell encapsulation and retrieval. It is characterized by blocks of (1–4)-linked β-d-mannuronic acid (M) and α-l-guluronic acid (G) monomers, showing structural similarities to hyaluronic acid (Park et al., 2009). The behavior of cells embedded in alginate is mainly influenced by its mechanical properties, which can be modified via addition of extracellular matrix components, such as fibronectin (Huang et al., 2012; Bozza et al., 2014; Andersen et al., 2015).

In this study, we report a 3D cell culture method based on hiPSC microencapsulation in small alginate/fibronectin beads that allows their efficient differentiation into functional DA neurons. Optimization of hydrogel matrix composition and concentration resulted in high viability of embedded hiPSCs and improved neural differentiation capacity to increase DA neuronal yield as compared to conventional 2D cultures. Furthermore, neurons grown in alginate scaffolds are characterized by increased electrophysiological functionality and maturity detected by elevated marker expression and an anticipated metabolic resetting.

## Materials and Methods

### Cell Lines

Human iPSCs from two control individuals (iPS-802 and iPS-SFC084-03-02) and one PD patient carrying a heterozygous triplication of the *SNCA* gene (iPS-ND34391) were used. iPSC-802 was generated in the laboratory of the Institute for Biomedicine by using a protocol we have published previously (Meraviglia et al., 2015). The control iPSC line SFC084-03-02 was established through the StemBANCC consortium.^1^ iPSC-ND34391 was obtained by the Coriell Cell Repository. The study was approved by the Ethics Committee of the South Tyrolean Health Care System (approval number 102/2014 dated 26.11.2014 with extension dated 19.02.2020).

### Human iPSC Culture

Human iPSCs were cultured under feeder-free conditions on Matrigel matrix (Corning) in StemMACS iPS-Brew XF (Miltenyi Biotech) with 1% penicillin-streptomycin (Thermo Fisher Scientific). Cells were maintained in a saturated humidified atmosphere at 37°C and 5% CO_2_, and medium was changed every day. hiPSC colonies were passaged enzymatically using 1 mg/ml Collagenase type IV (Thermo Fisher Scientific) in DMEM/F12 (Thermo Fisher Scientific).

### Encapsulation of hiPSCs

Alginate (Alg) solutions (1 and 2% w/v) were prepared by mixing alginic acid sodium salt (Sigma Aldrich) in a 0.025 M HEPES and 0.15 M NaCl buffer (pH 7.4). Fibronectin (Fn, #F2006, Sigma Aldrich) was added to the Alg at a final concentration of 100 μM. The day of cell encapsulation, 10^6^ hiPSCs were gently mixed with 1 ml Alg solution (1 or 2% w/v) with or without Fn. Alg beads were formed by slowly dripping the prepared Alg-cell suspension in 0.1 M CaCl_2_ with a 19-gauge needle, beads were then incubated for 20 min in the CaCl_2_ solution and washed 3 times with 1X PBS pH 7.4 (Thermo Fisher Scientific). Subsequently, cell-loaded beads were transferred to 12-well cell culture plates (10–12 beads/well) and supplied with StemMACS iPS-Brew XF medium supplemented with 10 μM Rho-associated protein kinase inhibitor (Y-27632, RI) (Miltenyi Biotech).

### Dopaminergic Neuronal Differentiation in 2D and 3D Conditions

For 2D monolayer differentiation, 3 × 10^4^ cells/cm^2^ were seeded on 12-well cell culture plates coated with Matrigel (Corning) in StemMACS iPS-Brew XF medium supplemented with 10 μM RI, and the differentiation of hiPSCs into midbrain DA neurons was initiated once the cells reached 90% confluence.

The differentiation with both the 2D and 3D methods was based on the floor-plate-based neural induction protocol established by Kriks et al., with minor modifications (Kriks et al., 2011; Zanon et al., 2017). In brief, differentiation was started by supplying the cells with knockout serum replacement (KSR) medium supplemented with SMAD pathway inhibitors SB431542 (SB, Tocris Bioscience) and LDN-193189 (LDN, StemMACS). LDN was added on days 1–10 while the supplementation with SB was stopped upon day 6. On days 2–7, recombinant Human Sonic Hedgehog (SHH, R&D System), recombinant Human FGF-8a (FGF8, R&D System) and Purmorphamine (Pu, StemMACS) were added to the medium. Furthermore, on days 4–12, the Wnt pathway activator molecule CHIR99021 (CH, StemMACS) was added to the differentiation medium. During days 7–10 of differentiation, increasing amounts of Neurobasal medium (Life Technologies) plus B27 supplement (Life Technologies) were added to the KSR medium (25, 50, and 75%). On day 12, maturation of DA neurons was initiated by adding recombinant Human BDNF (Peprotech), recombinant Human GDNF (Peprotech), ascorbic acid (AA, Sigma), recombinant Human TGF-beta 3 (β3, Peprotech), dibutyryl-cyclic-AMP (dbcAMP, EnzoLifescience) and DAPT (Tocris). On day 20 of differentiation, cells differentiated in 2D were passaged *en bloc* or as single cells on 24 well plates coated with Poly-D-lysine (Sigma Aldrich) and Laminin (Sigma Aldrich) (PDL/LA).

### Decapsulation of Differentiated Cells From Alginate Beads

Prior to decapsulation, Alg beads were washed inside the well with PBS without Ca^2+^ and Mg^2+^. Subsequently, the beads were distributed to 1.5 ml Eppendorf tubes (3–4 per tube) with PBS without Ca^2+^ and Mg^2+^ and shaken at 37°C and 700 rpm for 20 min. Then, cells were centrifuged at 300 x g for 5 min and collected for gene and protein expression or seeded at an appropriate density on cell culture plates with coverslips coated with 1 mg/ml Matrigel for immunofluorescence and functional analyses.

### Cell Viability Assay

The Live/Dead cell viability assay (Live/Dead cell viability assay kit, Thermo Fisher Scientific) was performed at days 5, 10, and 20 of differentiation. Single beads were placed in a live imaging chamber and incubated for 20 min with a 2 μM Ethidium Homodimer-1 (EH-1) and 8 μM Calcein (AM) solution in PBS in dark conditions. Images were acquired using a Leica SP8-X confocal microscope (Leica Microsystems) and analyzed using Imaris software (Oxford Instruments).

### RNA Extraction, RNA-Sequencing and Bioinformatics Analysis

Cells were recovered from the beads at days 10, 20, and 35 of differentiation, and total RNA was isolated using the Direct-zol RNA Miniprep Kit (#R2052, Zymo research). RNA integrity was assessed on an Experion Electrophoresis Station (Bio-Rad). The targeted RNA-sequencing libraries were produced using the DriverMapT Human Genome-Wide Expression Profiling Kit, V2 (Cellecta), which measures all 19,000 human protein-coding genes. All samples were sequenced in 38 paired-end reads on the Illumina NextSeq500 platform. Fold change of the eight genes shown in Figure 2B and Supplementary Figure 2B was calculated as follows. Raw reads (fastq) were checked using FastQC (v 0.11.9)^2^ and MultiQC (v 1.09) (Ewels et al., 2016). Transcripts were quantified with salmon (v 1.4.0) (Patro et al., 2017) on the human transcriptome GRCh38 (Ensembl 100). Differential expression analysis was performed with DESeq2 package (v 1.28.1) (Love et al., 2014) using the Wald test for significance testing. Principal component analysis was run with plotPCA function included in the DESeq2 package. Samples euclidean distance was calculated with the dist function and plotted with pheatmap (v 1.0.12) with complete clustering method. Three replicates per time point (hiPSC line, days 10, 20, and 35 of differentiation) and condition (2D and 3D) were used in the RNA-sequencing analysis on the SFC084-03-02 line. The complete RNA-sequencing experiment is available at GEO accession number GSE178683.

**FIGURE 1:**
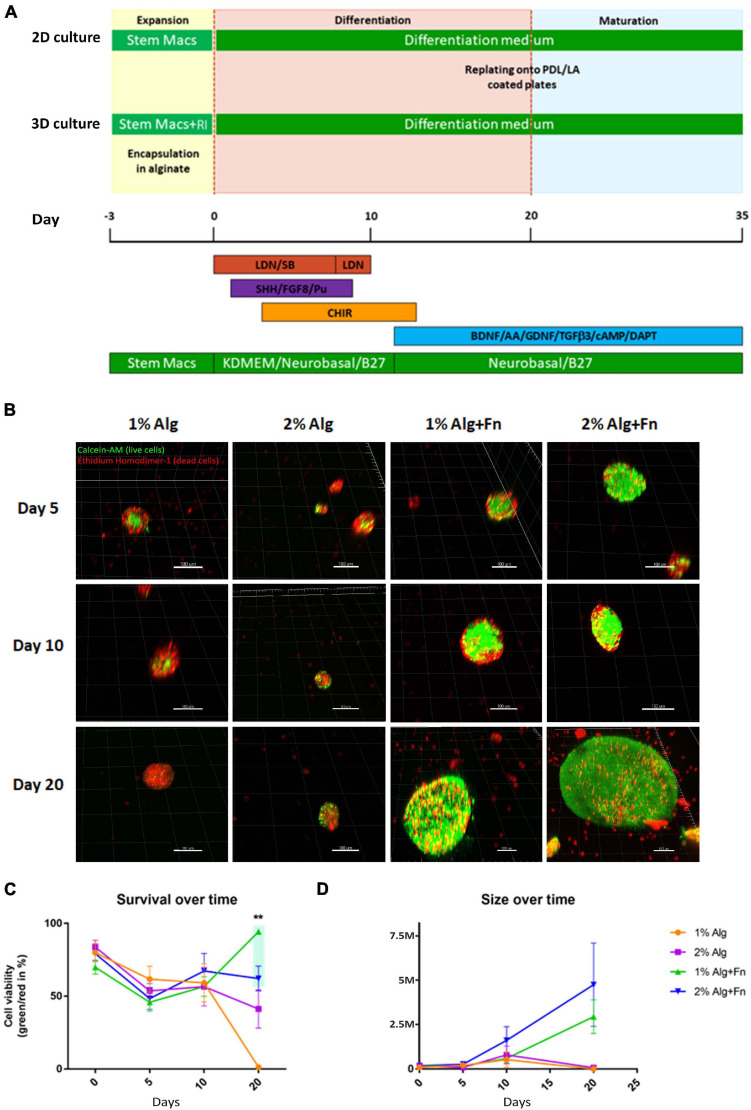
Viability of hiPSCs encapsulated and differentiated in alginate of different compositions.**(A)** The induction of early neuroectoderm differentiation from hiPSCs was achieved by dual SMAD inhibition, followed by the activation of SHH, Wnt, and FGF8 signaling pathways for patterning the midbrain fate. The committed neural progenitor cells were terminally differentiated into DA neurons by withdrawal of key neurogenic factors (BAGTCD). SM, StemMACS medium; RI, Rho associated kinase inhibitor; BAGTCD, BDNF, L-Ascorbic Acid, GDNF, TGFβ3, dbcAMP, DAPT. **(B)** Representative 3D reconstructions of cell aggregates stained with Calcein-AM (green, live cells) and Ethidium Homodimer-1 (red, dead cells). Scale bar represents 100 μm. **(C)** Cell viability over time was calculated as a percentage of green/red ratio. **(D)** Average size of the cell aggregates formed by viable cells was measured in millions of cubic micrometers. Statistical differences were calculated by two-way ANOVA followed by Tukey’s *post hoc* test to correct for multiple comparisons **p ≤ 0.005.

**FIGURE 2:**
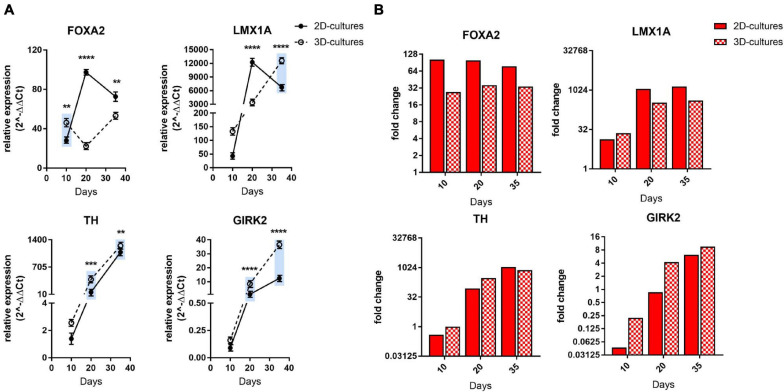
Comparative gene expression analyses of mDA progenitor induction as well as maturation into post-mitotic mDA neurons. **(A)** qRT-PCR analysis of FOXA2, LMX1A, TH, and GIRK2 at days 10, 20, and 35 of differentiation. The blue bars are illustrating an ameliorated phenotype of 3D cultures compared to the 2D counterpart. Statistical differences were calculated by multiple unpaired *t*-tests **p ≤ 0.01, ***p ≤ 0.001, ****p ≤ 0.0001. **(B)** Bars represent expression fold change of FOXA2, LMX1A, TH and GIRK2 genes at each time point (day 10, 20, 35) with respect to differentiation start (hiPSCs) in the RNA-sequencing experiment. Values below 1 indicate down-regulation of expression. Red bars display gene expression change in 2D culture, whereas bars with red and white checker pattern visualize those from 3D culture. A single fold change value is derived from three replicates for each comparison presented here.

### Quantitative Reverse-Transcription PCR (qRT-PCR)

RNA was reverse-transcribed using the SuperScript^®^ VILO^TM^ cDNA Synthesis Kit (Thermo Fisher Scientific) following the manufacturers’ protocol. qRT-PCR was performed in triplicate on the 96CFX Manager (Bio-Rad) in 20 μl using the All In One qPCR mix (Genecopoeia) and 0.2 μM of primers (Supplementary Table 1). The gene expression levels were normalized to the housekeeping gene β-actin using the ΔΔCt method.

### Western Blot Analysis

Protein lysate was loaded on a NuPAGE 4–12% Bis-Tris Gel (Thermo Fisher Scientific). After electrophoresis, proteins were transferred onto a nitrocellulose membrane (Bio-Rad) and subsequently incubated with the following primary antibodies: mouse anti-FOXA2 (#sc374376, Santa Cruz, 1:500), rabbit anti-LMX1A (#ab10533, Millipore, 1:1,000), mouse anti-TUJ1 (#MMS-435P, Biolegend, 1:1,000), rabbit anti TH (#657012, Calbiochem, 1:1,000), mouse anti-GAPDH (#MAB374, Santa Cruz, 1:1,000). Image Lab (Bio-Rad) was used for densitometric analysis of the blots.

### Immunofluorescence Staining

Cells differentiated by the 2D and 3D approaches were replated on days 10, 20, and 35 of differentiation. Cells were fixed in a 4% paraformaldehyde solution in PBS for 15 min at room temperature. Then, cells were permeabilized for 5 min with 0.5% Triton X-100 in PBS, blocked for 1 h with 3% BSA in PBS at room temperature and incubated overnight in 3% BSA at 4°C with the following primary antibodies: mouse anti-Nestin (#A24354, Thermo Scientific, 1:50), rabbit anti-PAX6 (#A24354, Thermo Scientific, 1:50), mouse anti-FOXA2 (#sc374376, Santa Cruz 1:50), rabbit anti-LMX1A (#AB10533, Millipore, 1:1,000), mouse anti-TH (#MAB318, Millipore, 1:500), rabbit anti-TH (#657012, Millipore, 1:200), goat anti-GIRK2 (#ab65096, Abcam, 1:100), rat anti-DAT (#sc-32259, Santa Cruz, 1:50), mouse anti-TUJ1 (#MMS-435P, Biolegend, 1:1,000), chicken anti-MAP2 (#ab5392, Abcam, 1:1,000), mouse anti-PSD95 (#36233S, Cell Signaling, 1:50), rabbit-anti Synapsin 1 (#52975, Cell Signaling, 1:100). For FOXA2 and LMX1A co-stainings, blocking and primary antibody incubation was conducted in PBS with 3% BSA and 0.05% Triton X-100. For TH and DAT as well as TH and GIRK2 co-stainings, cells were directly blocked in 10% FBS in PBS without permeabilization, and primary antibody incubation was performed in 5% FBS in PBS. Finally, coverslips were mounted using ProLong Diamond Antifade Mountant with DAPI (Thermo Fisher Scientific). Images were acquired using a Leica SP8-X confocal microscope (Leica Microsystems) and analyzed using Imaris software (Oxford Instruments).

### Live-Cell Imaging of Mitochondrial Function

Mitochondrial superoxide detection was performed by incubating the hiPSC-derived-neurons in HBSS containing 2.5 μM MitoSox Red mitochondrial superoxide indicator (Thermo Fisher Scientific) for 15 min at 37°C and 5% CO_2_. After incubation, cells were washed three times with HBSS. Mitochondrial membrane potential was measured using 50 nM TMRM mitochondrial membrane potential indicator (Thermo Fisher Scientific) for 30 min at 37°C and 5% CO_2_. Mitochondrial morphology was determined by staining the cells with 1 μM MitoTracker Green FM (Thermo Fisher Scientific) for 15 min at 37°C and 5% CO_2_. Images were acquired in live using a Leica SP8-X confocal microscope (Leica Microsystems) and analyzed using Imaris software (Oxford Instruments).

### Vibratome Sectioning of Alginate Cell Aggregates

Cell aggregates of DA neurons differentiated in 1% Alg-Fn for 200 days were fixed with 4% paraformaldehyde overnight at 4°C, washed three times with 1X PBS and embedded in 3% agarose. The solid agarose block was sectioned into 80 μm slices using a Leica VT1000 S vibratome (Leica Biosystems). The sections were stored in 1X PBS at 4°C until use for immunofluorescence staining.

### Electrophysiological Characterization

Whole-cell recordings in current-clamp mode were performed in a temperature-controlled recording chamber (36–37°C) mounted on an inverted Eclipse-Ti microscope (Nikon) and using a MultiClamp 700B amplifier (Molecular devices, LLC). Current-command protocols and data acquisition were performed using pClamp 10.0 software and the Digidata 1550 interface (Molecular Devices, LLC). Data were lowpass-filtered at 3 kHz and sampled at 10 kHz. Patch electrodes, fabricated from thick borosilicate glass capillaries, have been made using a Sutter P-1000 puller (Sutter Instruments) to a final resistance of 4–6 MΩ when filled with the intracellular solution containing (in mM): 120 KGluconate, 25 KCl, 10 EGTA, 10 HEPES, 1 CaCl_2_, 4 Mg-ATP, 2 Na-GTP, and 4 Na_2_-Phosphocreatine (pH 7.4, adjusted with KOH). Cells were bath-perfused with a Krebs solution composed of (in mM): 129 NaCl, 5 KCl, 2 CaCl_2_, 1 MgCl_2_, 30 D-glucose, 25 HEPES, pH 7.3 with NaOH. Spontaneous action potentials were recorded in a gap-free mode while the presence of the Ih current was evaluated by applying long hyperpolarizing steps at different current intensities (−30 pA increments). Series resistance value was monitored during the experiment, and recordings with changes over 20% of their starting value were discarded.

### Statistical Analyses

Statistical analyses were performed using GraphPad Prism 8. One-way ANOVA was used in experiments comparing at least three groups, followed by Tukey’s or Bonferroni *post hoc* test for pairwise comparisons. For analyzing differences between two experimental groups, the unpaired two-tailed Student’s *t*-test was utilized. Threshold for significance was set at *p* < 0.05. All experiments were performed in at least three independent biological replicates, except for the analyses presented in Figure 5, for which we used two independent replicates.

**FIGURE 3:**
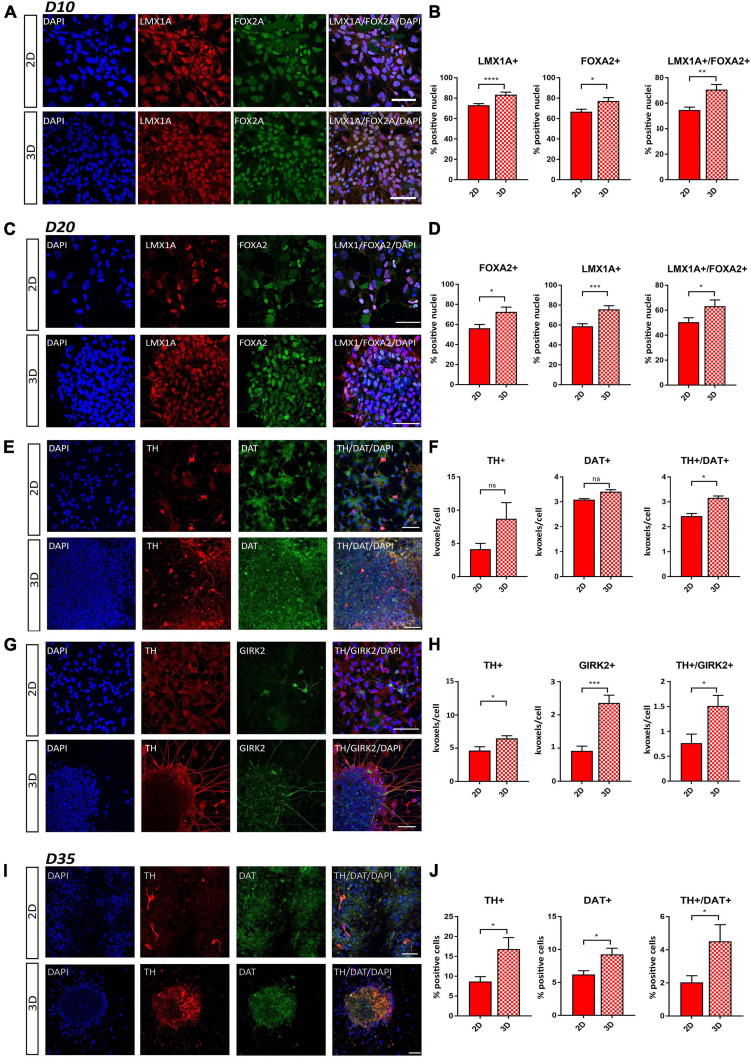
Comparative immunofluorescence analysis of early DA neuron markers. **(A)** Representative staining of LMX1A^+^ and FOXA2^+^ cells at day 10 of differentiation. **(B)** Percentage of LMX1A^+^, FOXA2^+^, and LMX1A^+^/FOXA2^+^ double positive cells at day 10 of differentiation. **(C)** Representative staining of LMX1A^+^ and FOXA2^+^ cells at day 20 of differentiation. **(D)** Percentage of LMX1A^+^, FOXA2^+^, and LMX1A^+^/FOXA2^+^ double positive cells at day 20 of differentiation. **(E)** Representative staining of TH^+^ and DAT^+^ cells at day 20 of differentiation. **(F)** Quantification of TH^+^, DAT^+^, and TH^+^/DAT^+^ double positive cells at day 20 of differentiation by using positive volume (thousands of voxels, kvoxels) divided by the number of nuclei. **(G)** Representative staining of TH^+^ and GIRK2^+^ cells at day 20 of differentiation (for this combination, another TH-antibody was used as in **E**). **(H)** Quantification of TH^+^, GIRK2^+^, and TH^+^/GIRK2^+^ double positive cells at day 20 of differentiation by using positive volume (thousands of voxels, kvoxels) divided by the number of nuclei. **(I)** Representative staining of TH^+^ and DAT^+^ cells at day 35 of differentiation. **(J)** Quantification of TH^+^, DAT^+^, and TH^+^/DAT^+^ double positive cells at day 35 of differentiation by counting the number of positive cells, which were clearly identifiable at this stage. Scale bars represent 50 μm. Statistical differences were calculated by a paired two-tailed *t*-test *p ≤ 0.05, **p ≤ 0.01, ***p ≤ 0.001, ****p ≤ 0.0001.

**FIGURE 4:**
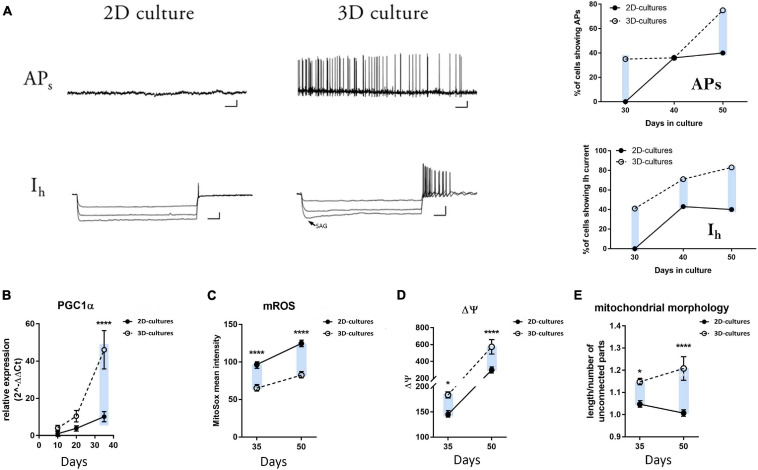
Electrophysiological and metabolic analyses of mDA neuron maturation in the 2D and 3D cultures. **(A)** Representative traces of whole-cell patch clamp recordings of hiPSC-derived neurons in 2D and 3D cultures at day 30. The arrow for the hyperpolarization-activated cation current (Ih) in the 3D cultures indicates the voltage-sag response upon hyperpolarizing current injections characteristic of the HCN channel activation. Scale bars APs: 10 mV/4 sec; Ih: 20 mV/200 ms. No spontaneous or evoked APs and no Ih could be recorded for the neurons cultured in 2D (*n* = 0/14 neurons). Plots on the right display the percentage of cells showing spontaneous APs and the presence of Ih at days 30, 40, and 50 during neuronal differentiation. **(B)** qRT-PCR analysis of PGC1-α at days 10, 20, and 35 of differentiation. **(C)** mROS production, **(D)** mitochondrial membrane potential, and **(E)** mitochondrial morphology in the differentiating neurons at days 35 and 50. The blue bars are illustrating an ameliorated phenotype of 3D cultures compared to the 2D counterpart.

**FIGURE 5:**
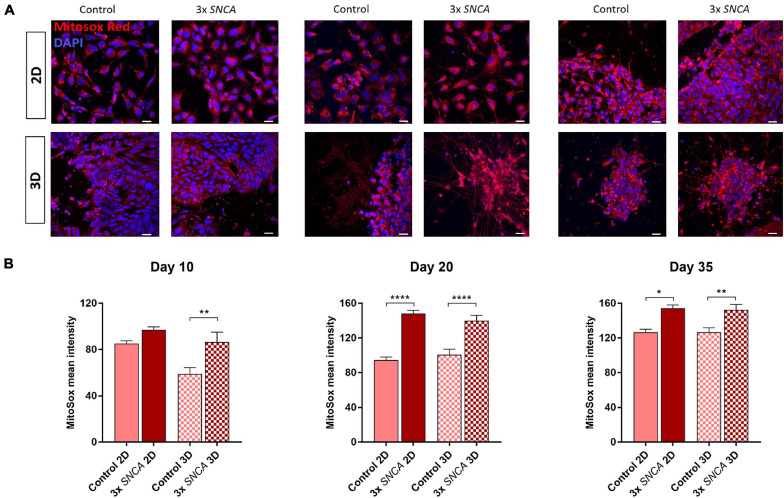
Mitochondrial superoxide levels (mROS) quantified with the fluorogenic dye MitoSox Red in 2D and 3D cultures. **(A)** Representative fluorescence images at different time points. **(B)** Quantification of MitoSox intensity (mean ± SEM) in DA neurons differentiated from a control and a PD patient (3x *SNCA* mutant) hiPSC line at days 10, 20, and 35 during neuronal differentiation. Data are derived from two independent experiments. Scale bars represent 50 μm. Statistical differences were calculated by one-way ANOVA followed by Tukey’s *post hoc* test to correct for multiple comparisons *p ≤ 0.05, **p ≤ 0.01, ****p ≤ 0.0001.

## Results

### Identification of Initial Culture Conditions and Hydrogel Composition to Best Sustain the Propagation and Viability of hiPSCs

Alginate (Alg) forms widely used hydrogels because it offers easy gelation in physiological conditions, lower batch-to-batch variations than animal materials, the possibility of gentle dissolution, transparency allowing microscopic observations, and the formation of a mesh allowing nutrient and waste diffusion (Andersen et al., 2015). Therefore, we attempted to identify a hydrogel composition (Alg with or without fibronectin, Fn) and initial culturing conditions, suitable to sustain cell-viability and propagation of hiPSCs, within a 3D Alg hydrogel matrix, that could mimic the elastic environment encountered in the developing brain.

It has been previously reported that Rho-associated protein kinase inhibitor (RI) is beneficial for viability and cell aggregation of hPSCs (Ohgushi et al., 2010; Horiguchi et al., 2014), and specifically it was shown to be essential for sustaining viability of hESCs in Alg microcapsules (Chayosumrit et al., 2010; Kim et al., 2013). Therefore, we also tested its beneficial effect for encapsulated hiPSCs. From the day of encapsulation in 1 or 2% Alg with or without Fn, control-hiPSCs were treated with RI for 3 days before neuronal differentiation, which was performed as outlined in Figure 1A. The proliferating cells formed aggregates within the hydrogel beads, which were counted manually. The number of aggregates in RI-treated Alg beads was significantly higher than in the non-treated beads, except for 2% Alg (Supplementary Figure 1A). Therefore, RI pre-treatment was used for all experiments.

To examine which Alg concentration would best support the survival and differentiation of the encapsulated hiPSCs, we tested 1 or 2% w/v. Both concentrations have been indicated as suitable for neuronal differentiation of pluripotent stem cells in previous publications (Wang et al., 2009; Li et al., 2011; Bozza et al., 2014), although Alg alone is thought to not provide adequate cell adhesion (Rowley et al., 1999). Therefore, we also tested both Alg concentrations modified by the addition of the glycoprotein Fn, a prominent extracellular matrix component, which is known to be involved in neural development, axon pathfinding and regeneration (Luckenbill-Edds, 1997; Perris and Perissinotto, 2000). The number of cell aggregates on the spherical Alg beads with a diameter of ∼400 μm, was counted at days 5, 10, and 20, and no significant differences were observed at any time point between 1 and 2% Alg. However, a significantly higher number of aggregates in both 1 and 2% Alg matrices containing Fn was observed compared to Alg alone (Supplementary Figures 1B,C). The highest number of aggregates was obtained for hiPSCs encapsulated in 1% Alg with Fn at day five of differentiation (*n* = 35 on average). At days 5 and 10, no difference in the dimension of aggregates was detected. At day 20, the average size of aggregates was higher in both 1% Alg with Fn and 2% Alg with Fn as compared to Alg alone. Furthermore, the average size of aggregates was higher in 2% than in 1% Alg with Fn (Supplementary Figure 1D). However, a good balance in the size of the aggregates is advantageous in order to avoid that too large aggregates escape from the beads.

In order to examine the viability of hiPSCs encapsulated in Alg with and without Fn, we performed a Live/Dead assay on the intact beads. In most cases, the Alg beads remained roughly spherical, and viability of cellular aggregates was evaluated at days 5, 10, and 20 of differentiation (Figure 1B). From days 0 to 20 of differentiation, the number of viable cells in 1 and 2% Alg with Fn was higher compared to Alg alone. In addition, at day 20, almost all cells encapsulated in 1% Alg alone died, and cell viability in 2% Alg beads was clearly decreased compared to the conditions with Fn (Figure 1C). Furthermore, we investigated the size of the aggregates formed by viable cells by determining the volume of aggregates by 3D reconstruction (Figure 1D). In line with the results obtained by bright field microscopy (Supplementary Figures 1B,D), we found that hiPSCs in 2% Alg with Fn formed the largest aggregates followed by the ones in 1% Alg with Fn. In both conditions, the aggregate size increased steadily over time. In contrast, the cell aggregates in 1 and 2% Alg alone did not increase in size during the examined differentiation period. Based on these data, we established 1% Alg with Fn as the most suitable experimental culturing condition, highlighting the crucial role of Fn in supporting cell viability during encapsulation and neuronal differentiation of hiPSCs.

### Comparative Molecular Analyses Suggest Enhanced DA Neuronal Differentiation in 3D

Next, we investigated differentiation into a midbrain dopaminergic (mDA) neuronal subtype by qRT-PCR, immunofluorescence, and western blot analyses at defined time intervals in 1% Alg with Fn. The combined expression of the transcription factors FOXA2 and LMX1A is essential for formation of a ventral midbrain identity (Arenas et al., 2015) and the *in vitro* generation of a floorplate-derived midbrain cell lineage (Kriks et al., 2011). Consistent with natural mDA development, both patterning markers were highly expressed during the entire differentiation period under both 2D and 3D conditions (Figure 2A). In the 2D mDA cell culture model, the expression of these markers decreased at day 35, whereas for the 3D mDA neurons, their expression levels remained more stable over time, as expected for the expression profile of developing mDA neurons. Interestingly, the expression of tyrosine hydroxylase (TH), the rate limiting enzyme in dopamine production, and of the G-protein-regulated inward-rectifier potassium channel 2 (GIRK2) were constantly higher during 3D differentiation when compared to the 2D culture, denoting a more rapid attainment of an mDA marker expression profile, and increased maturation (Figure 2A). Additionally, for the class III member of the beta tubulin family (TUJ1), which is involved in axon guidance and is frequently used as a neuronal marker (Tischfield et al., 2010), we could detect an increased expression level in the 3D compared to the 2D model during the entire differentiation process (Supplementary Figure 2A). We also analyzed the expression of the vesicular glutamate transporter 1 (vGLUT1) as a marker for glutamatergic neurons, glial fibrillary acidic protein (GFAP) for astrocytes, and glutamate decarboxylase 1 (GAD1) for GABAergic neurons and did not find a significant difference at day 10, while an increase in the expression levels of these three genes at a later stage during differentiation was observed for the 3D platform, suggesting a more complex and mature neuronal culture compared to the 2D system (Supplementary Figure 2A).

To further explore mDA progenitor induction as well as maturation into post-mitotic neurons, we also analyzed the transcriptional profile of these marker genes by RNA sequencing of the same control-hiPSC line (Supplementary Figures 2B,C). TH and GIRK2 expression patterns showed a similar trend across the two methods; for FOXA2 and LMX1A, a stable expression during differentiation was confirmed, although some differences to the qRT-PCR expression data were observed (Figure 2B). Furthermore, TUJ1 and GAD1 showed a very similar trend in the transcriptomics analysis, while vGLUT1 and GFAP showed differences at some time points (Supplementary Figure 2D).

To gain a deeper understanding of the ability of our matrix to sustain neural differentiation, immunofluorescence staining was conducted for well-defined early and mDA neuronal markers at days 10, 20, and 35 of differentiation. At day 10, a higher expression level of PAX6, an early neural commitment marker, was observed under the 3D conditions, indicating a more rapid attainment of an early developmental stage. No statistically significant difference in the expression of the neuronal progenitor marker Nestin could be observed (Supplementary Figures 3A,B). The analysis of the patterning markers LMX1A and FOXA2 showed that the percentages of LMX1A^+^ and FOXA2^+^ cells were significantly increased in the 3D cell culture conditions compared to cells generated using the 2D protocol. Additionally, the signal from double positive (LMX1A^+^/FOXA2^+^) neuronal cells was significantly increased in our 3D condition (Figures 3A,B). These data were confirmed also at day 20 of differentiation (Figures 3C,D), demonstrating an enhancement in the development of a floorplate-derived midbrain fate in the 3D model system.

To further characterize the extent of differentiation, we analyzed the co-staining of the DA neuronal marker TH together with the pan-neuronal marker TUJ1, the mDA neuronal maturation marker GIRK2, and the dopamine transporter (DAT) at day 20 of differentiation. We were able to clearly identify GIRK2^+^ and DAT^+^ neurons and observed a higher proportion of TH^+^ and GIRK2^+^ cells as well as TH^+^/DAT^+^ and TH^+^/GIRK2^+^ double positive cells under 3D conditions (Figures 3E–H). Additionally, in the 3D condition, we found a higher fraction of TUJ1^+^ and TH^+^/TUJ1^+^ double positive cells (Supplementary Figures 3C,D). In accordance with this, we observed an increase in the TH^+^ and DAT^+^ cells as well as the fraction of TH^+^/DAT^+^ double positive cells in the 3D neurons at day 35 of differentiation (Figures 3I,J).

Finally, we used Western blot analysis to quantify protein expression levels of mDA neuronal differentiation markers. We found higher levels of the transcription factors FOXA2 and LMX1A in the 3D model system, compared to 2D differentiated cells, at days 20 and 35 of differentiation (Supplementary Figures 3E,F). In line with our qRT-PCR and immunofluorescence data, we also observed a stronger upregulation of the expression of TUJ1 and TH (Supplementary Figures 3E,F).

### Electrophysiological Analyses Point to Higher mDA Neuron Maturation in the 3D Cultures

To functionally assess the mDA neuronal maturation of our 2D and 3D cultures, we performed electrophysiological recordings at days 30, 40, and 50 of differentiation. Individual neurons were recorded in whole-cell configuration to evaluate the presence of both spontaneous firing activity (APs) and the hyperpolarization-activated cation current (Ih). Endogenous human midbrain DA neurons display spontaneous rhythmic firing of action potentials from 2 to 10 Hz (Grace and Bunney, 1984; Ramayya et al., 2014). The Ih current is an inward depolarizing current mediated by the hyperpolarization-activated cyclic nucleotide-gated (HCN) cation channels, and it is a typical characteristic of mature mesencephalic DA neurons (Chu and Zhen, 2010). At day 30 of differentiation, 35% of the recorded hiPSC-derived neurons in the 3D cultures were able to generate both spontaneous as well as evoked APs with an average spontaneous firing rate of 2.7 ± 0.97 Hz (mean ± SEM; Supplementary Table 2). On the contrary, we could not record any APs for the neurons generated using the conventional 2D protocol at this stage of differentiation (Figure 4A and Supplementary Table 2). Additionally, 41% of the patched 3D neurons displayed the voltage-sag response upon hyperpolarizing current injections, indicating the presence of the HCN-mediated currents (Figure 4A and Supplementary Table 2). Again, none of the recorded 2D neurons at day 30 was characterized by the presence of these currents. Differences for these parameters were maintained during later stages of differentiation (days 40 and 50, Figure 4A and Supplementary Table 2). Moreover, we examined the passive electrophysiological properties of the differentiated neurons, which are inherent to the cell membranes and are able to modulate neuronal integration and firing patterns. The resting membrane potential (mV) was significantly lower at day 30, and membrane capacitance (pF) was significantly higher in the 3D neurons at days 30 and 50 as compared to 2D neurons (Supplementary Figure 4A). Active and passive electrical properties, exhibited by the neurons generated in the 2D and 3D cultures, are summarized in Supplementary Table 2. Overall, these data indicate that the DA neurons generated using the 3D system reached a relatively advanced functional level of *in vitro* maturity earlier during differentiation when compared to neurons generated using the conventional 2D culture system as indicated by pacemaking firing capacity and the functional expression of the Ih current.

### Metabolic Switching Profile Reveals an Earlier Metabolic Resetting During Neuronal Differentiation in the 3D Platform

Metabolic changes are specific hallmarks in directing cell fate in the nervous system. Stem cells are characterized by elevated rates of aerobic glycolysis and the absence of oxidative phosphorylation, which are both required for the maintenance of their pluripotency (Varum et al., 2011; Zhang et al., 2011; O’Brien et al., 2015; Gu et al., 2016; Zheng et al., 2016; Mathieu and Ruohola-Baker, 2017).

Several studies have highlighted that differentiation of PSCs induces a variety of mitochondrial morphological and functional changes, which allow mitochondrial maturation and bioenergetics transition from anaerobic to aerobic metabolism (Chung et al., 2007; Armstrong et al., 2010; Prigione et al., 2010; Tormos et al., 2011; Agostini et al., 2016; Maffezzini et al., 2020). PGC-1α is an essential regulator of mitochondrial biogenesis, known to play a key role in neuronal metabolism (Lin et al., 2004). We found a significant increase in the expression level of this gene in the 3D culture conditions at day 35 of differentiation compared to the standard 2D protocol, with a tendency for higher expression levels already at earlier time points, indicating an earlier and more pronounced shift to a post-mitotic neuronal status (Figure 4B). In addition, we measured mitochondrial superoxide (mROS) production, mitochondrial membrane potential, and mitochondrial morphology in the differentiating neurons at days 35 and 50. As highlighted by a number of reports for the transition of neural stem cells to their mature neuronal stage (Maffezzini et al., 2020), we observed significantly reduced levels of mROS at days 35 and 50 in the 3D hiPSC-derived neurons (Figure 4C and Supplementary Figure 4B). Accordingly, 3D neurons exhibit higher levels of TMRM fluorescence, which indicates a higher mitochondrial membrane potential (Figure 4D and Supplementary Figure 4C). Finally, the mitochondrial morphology appeared more elongated for the 3D cultures at both time intervals (Figure 4E and Supplementary Figure 4D). These data suggest the occurrence of an earlier metabolic switch in the 3D neurons compared to their 2D counterparts, further supporting a faster differentiation and maturation of the hiPSCs inside the 3D Alg matrix.

### Molecular Analyses of Long-Term mDA Neural Differentiation in 3D Cultures

Performing the long-term cultures of differentiated DA neurons could be a useful approach to recapitulate the aging process *in vitro*. This is a challenging endeavor considering that neurons, cultured for long periods, tend to lose viability. We were able to keep DA neurons differentiated both in 2D or encapsulated in 1% Alg/Fn in culture for 200 days. The gene expression of the mature DA marker proteins DAT, TH and GIRK2, as well as the pan-neuronal marker TUJ1 was determined by qRT-PCR. We observed a significant increase in the expression levels of TH, GIRK2, and DAT in the 3D neurons in comparison to 2D neurons (Supplementary Figure 4E).

Immunofluorescence staining on sections of Alg capsules shows that both DA marker proteins DAT and TH are expressed. The stained areas are partially overlapping indicating co-expression of both proteins. The most intense staining for both proteins was observed in the margins of the sections, which could be due to a higher DA neuron density overall or specifically in the periphery of the cell aggregates (Supplementary Figure 4F). By evaluating the expression of the presynaptic marker protein Synapsin 1 (Syn1) and the postsynaptic density protein 95 (PSD95), we also showed the formation of multiple synaptic connections in the neurons within the aggregates. While the Syn1 signal was located in neurites and possibly cell bodies, the PSD95 staining demonstrated a punctated pattern, indicating a more confined expression. In order to confirm neuronal identity, we co-stained the sections for the neuronal marker microtubule associated protein 2 (MAP2). In a 3D surface reconstruction, we illustrated the juxtaposed signals of Syn1 and PSD95 staining, which might denote the presence of pre- and post-synaptic puncta and multiple synaptic connections (Supplementary Figure 4G).

### 3D Alginate Model System Recapitulates High mROS Levels in 3xSNCA Mutation Carrier

Oxidative stress plays an important role in the cellular lesions that may lead to cell death (Hemmati-Dinarvand et al., 2019; Chang and Chen, 2020). The generation of ROS correlates with mitochondrial dysfunction, which is a widely accepted pathogenic mechanism implicated in PD (Lin and Beal, 2006; Dias et al., 2013). Increased oxidative stress is also a well-known feature of hiPSC-derived neurons with mutations in PD-related genes, like *SNCA*, encoding alpha-synuclein (Byers et al., 2011; Imaizumi et al., 2012; Flierl et al., 2014). We compared mROS generation in DA neurons differentiated in 2D and 3D culture at days 10, 20, and 35 in a control line and a patient line carrying an alpha-synuclein triplication (3x*SNCA*) mutation. The patient line showed higher mROS levels compared to the control line under both 2D and 3D conditions. However, in the 3D Alg cultures, the aberrant phenotype was observed already at day 10 of differentiation (Figure 5). These findings indicate that our 3D model system can recapitulate an important mitochondrial phenotype observed in neurons derived from PD patients, and that this phenotype might be detectable earlier during neuronal differentiation.

## Discussion

The advent of cellular reprogramming and iPSC-technology in combination with directed differentiation protocols made it possible to generate patient-specific and disease-affected cell types *in vitro*. Most *in vitro* neurodegenerative disease modeling studies so far are based on conventional 2D cell culture systems, which show a variable reproducibility with relatively low degrees of functional maturation, whereas 3D cell culture models are thought to better mimic cell growth encountered *in vivo* and support the expression of tissue-specific genes and proteins (Geckil et al., 2010). Therefore, we investigated the potential of an Alg-based 3D culture system to support the generation of mature and functional hiPSC-derived mDA neurons for PD modeling in comparison to an established 2D protocol. We identified a biomaterial combination that supported the survival of hiPSCs embedded in Alg beads and their differentiation to mDA neurons. By using expression analyses, electrophysiological and metabolic measurements, we demonstrated a higher functional maturity of our 3D model and found that it was able to recapitulate a known mitochondrial phenotype. Furthermore, we showed that our 3D cultures are suitable for long-term culture of hiPSC-derived DA neurons.

The native extracellular matrix (ECM), in which cells are embedded, is an important part of their extracellular microenvironment, which profoundly influences tissue organization but can also be seen as a cellular extension and active participant in the regulation of cellular function (Rozario and DeSimone, 2010; Nicolas et al., 2020). In fact, 3D cultures, where these factors can be modeled to a certain extent, are more closely resembling the *in vivo* counterparts than cells cultured in 2D, which do not express certain cell-specific markers at levels comparable to those found *in vivo* (Pampaloni et al., 2007; Geckil et al., 2010). Furthermore, cells grown in 3D in several studies showed improvements for biological mechanisms like viability, morphology, proliferation, differentiation, response to stimuli, and drug metabolism (Antoni et al., 2015; Jensen and Teng, 2020). Brain ECM is characterized by a complex network of polysaccharide-protein complexes called proteoglycans, mostly formed by hyaluronic acid interaction with a variety of proteins (Ruoslahti, 1996; Mouw et al., 2014). Hyaluronic acid as a native brain ECM component requires chemical modification to allow its incorporation into a hydrogel suitable for cell culture applications. However, brain ECM can partially be modeled by natural hydrogels, 3D cross-linked hydrophilic, insoluble polymers, such as Alg and Fn. To obtain a hydrogel, the individual linear Alg polymer chains have to be crosslinked, most commonly by ionic crosslinking (Lee and Mooney, 2012), which consists in the exposure of the unmodified Alg to divalent cations, such as Ca^2+^, as performed in this study. Cell behavior is mainly influenced by mechanical properties of Alg, such as viscosity, stiffness and degradability, which can be shaped by altering concentration, gelation procedure and the addition of ECM components such as the glycoprotein Fn (Andersen et al., 2015). Fn is a V-shaped protein consisting of two nearly identical subunits joined by a pair of disulfide bonds, which interconnects ECM components with each other and with cells. It has binding sites for both collagen and glycosaminoglycans, like hyaluronic acid, which enable it to crosslink these ECM components and facilitates the linking of ECM components to cells (Walker et al., 2018). Additionally, Fn is used for surface coating in 2D monolayer cultures, but in this form, it is unable to recapitulate the complex neural ECM network. Hydrogels based on Alg can be dissolved, and cells can easily be recovered by chelating agents such as EDTA and phosphate, or non-gelling agents such as sodium or magnesium ions (Huang et al., 2012).

Previously, Alg/Fn hydrogels have been used already for the culture of mESCs and their differentiation to neural lineages (Bozza et al., 2014) but had not been explored yet for the culture of hiPSCs and their differentiation to mDA neurons. Our data show that the encapsulation of hiPSCs in both 1 and 2% Alg results in the formation of viable cell aggregates. The higher number of aggregates in 1% Alg, supplemented with Fn, reflects an increased cell survival after encapsulation under this condition. The decreasing number of cell aggregates observed over time may be explained by several factors: growing aggregates may fuse with others or impede the counting of others underneath, and aggregates in the periphery occasionally grow out of the Alg beads and are therefore not included in the analysis. It has been shown that Rho-associated protein kinase inhibitor (RI) is a crucial factor for the sustainment of hESC viability during the first three days after encapsulation in Alg (Chayosumrit et al., 2010; Kim et al., 2013). We showed that the treatment with RI is essential also for the initial survival of hiPSCs embedded in this biomaterial as very few cell aggregates were observed after encapsulation without RI treatment. Based on the number and viability of the formed aggregates, we identified 1% Alg modified with Fn as the most suitable scaffold composition for the culture and differentiation of hiPSCs in 3D.

The cellular 3D microenvironment is an important determinant of gene expression, which in turn influences cellular identity and function (Engler et al., 2006; McKee and Chaudhry, 2017). For this reason, we compared the gene expression patterns of neurons differentiated in 1% Alg/Fn to neurons differentiated in 2D. Overall, qRT-PCR, immunofluorescence and western blot analyses resulted in higher expression levels of both neuronal commitment and mature DA neuron markers in the 3D condition. The co-expression of the transcription factors LMX1A and FOXA2 is essential for the specification of DA neurons (Engler et al., 2006; Arenas et al., 2015), and its importance for the generation of high quality mDA neurons *in vitro* has been shown (Kriks et al., 2011). Consistent with natural mDA development, in the 3D condition, these markers increased their expression levels over time in the mDA neurons, whereas in the 2D cell culture system the expression was higher in some instances but declined at day 35 of differentiation, as shown by the qRT-PCR analyses. TH and GIRK2 protein levels are crucial for DA neuronal function and commonly used as markers for mature DA neurons. We were able to show consistently higher expression and co-expression of both markers in neurons cultured in 1% Alg/Fn as compared to 2D. Furthermore, DAT expression and its co-expression with TH was increased in the 3D cultures. Thus, the stable expression of all these markers, known to specifically regulate the development of A9-subtype ventral mDA neurons, indicates the effective generation and maintenance of a *substantia nigra* specific midbrain fate in the 3D Alg neurons.

The expression analysis of specific mature DA marker proteins is an important way to assess the success of differentiation toward mDA neurons but the broader goal is the generation of fully functional excitable mDA neurons. Indeed, electrophysiological recordings identified a significant proportion of 3D DA neurons able to generate spontaneous APs and Ih currents already at day 30, while no spontaneous APs and Ih currents could be recorded in 2D cultures at this time point. Significant differences for these parameters were maintained during later stages of differentiation. The measured average firing rate of the recorded cells was within the range reported for mDA neurons (Grace and Bunney, 1984; Hartfield et al., 2014; Ramayya et al., 2014). These data indicate that the 3D culture system might better support the generation of mature functional mDA neurons, which is further emphasized by the recorded passive electrophysiological properties. These results are further supported by an anticipated metabolic resetting during neuronal differentiation in 3D Alg/Fn cultures. In order to examine the utility of our 3D model system to recapitulate known PD-related phenotypes, we chose to investigate mROS production in a 3D differentiated α-synuclein triplication (3x*SNCA* mutation) line in comparison to a control line. The mROS levels were significantly higher in the 3x*SNCA* mutant neurons cultured under 3D conditions already at day 10, suggesting that the increased mROS phenotype of PD patient neurons might be detectable earlier during neuronal differentiation in the 3D model system. Furthermore, we were able to show that hiPSC-derived DA neurons could be sustained for at least 200 days in the 3D culture system, circumventing the problem of cell detachment often observed upon long-term culture in 2D. We showed that mDA neuron marker expression was still increased at this advanced time point and confirmed the presence of synaptic connections formed by 3D neurons by immunofluorescence stainings.

Our Alg-based 3D culture system is not directly comparable to established midbrain-specific organoids (Jo et al., 2016; Monzel et al., 2017; Smits et al., 2019). The size of the organoids, which is in the range between one and several mm, represents the first substantial difference to our cell aggregates that reach a length of about 400 μm at day 20 of differentiation. Furthermore, each Alg bead contains several cellular aggregates. Due to the size of organoids, diffusion-dependent nutrient supply and waste removal is not efficient, leading to necrosis of the inner core, which can only partially be solved by shaking cultures (Lancaster et al., 2013; Qian et al., 2016). This drawback is not present for the neuronal aggregates in the Alg beads. Additionally, the starting point of our 3D Alg aggregates are single hiPSCs, while organoids are often started from colonies of PSCs or pre-specified neural stem cells embedded in an extracellular matrix and placed in a spinning reactor, where they self-organize into structures with compartments corresponding to different brain regions (Lancaster et al., 2013). The development of these organoids is dependent on the stochastic nature of the differentiation processes, which leads to organoids with a high degree of heterogeneity in terms of maturity and functionality. Our 3D neuronal aggregates are not aimed at reproducing the tissue-specific neural composition and architectural organization that is characteristic for organoids, but they are rather intended as a system to generate functional mature neurons in an efficient and robust manner. Finally, while Alg 3D neurons can be easily recovered from the microcapsules for downstream functional analysis and are therefore also suitable for drug screening approaches, relevant high-throughput measurements for drug screening applications in organoids need the integration of miniature biosensors, limiting their translatability.

In summary, the Alg/Fn 3D scaffold promoted hiPSC viability and growth and allowed for an optimized differentiation of hiPSCs to mDA neurons as a complementary approach to the generation of midbrain-specific organoids. The characteristics of the Alg/Fn mDA neuron cultures include an increased expression of neurogenesis and neural maturation markers accompanied by an increased electrophysiological functionality and an earlier metabolic resetting profile as compared to neurons grown in monolayer cultures. Collectively, these data indicate that the Alg/Fn 3D scaffold represents a tool for reliable and rapid derivation of more mature and functional mDA neurons from hiPSCs, demonstrating the experimental advantages of using a 3D culture system over more traditional 2D cultures.

## Data Availability Statement

The datasets presented in this study can be found in GEO online repository. The name of the repository and accession number can be found in the article.

## Ethics Statement

The studies involving human participants were reviewed and approved by the Ethics Committee of the South Tyrolean Health Care System (approval number 102/2014 dated 26.11.2014 with extension dated 19.02.2020). The patients/participants provided their written informed consent to participate in this study.

## Author Contributions

VG, GG, DR, and MV carried out the experiments. AL performed the imaging analyses. EK and CW performed transcriptomic analyses. MR-S carried out the electrophysiology experiments and analyses. SC, LC, PP, and AH contributed to the final version of the manuscript. IP discussed the results, wrote the manuscript, and coordinated the project. AZ conceived the original idea, designed the experiments, wrote the manuscript, and supervised directly the project. All authors contributed to the article and approved the submitted version.

## Conflict of Interest

The authors declare that the research was conducted in the absence of any commercial or financial relationships that could be construed as a potential conflict of interest.

## Publisher’s Note

All claims expressed in this article are solely those of the authors and do not necessarily represent those of their affiliated organizations, or those of the publisher, the editors and the reviewers. Any product that may be evaluated in this article, or claim that may be made by its manufacturer, is not guaranteed or endorsed by the publisher.

## Footnotes

1
https://cells.ebisc.org/STBCi033-B/

2
http://www.bioinformatics.babraham.ac.uk/projects/fastqc/
