# Supplementary material for: Generation of hiPSC-Derived Functional Dopaminergic Neurons in Alginate-Based 3D Culture
Source: Front Cell Dev Biol. 2021 Aug 2;9:708389. doi: 10.3389/fcell.2021.708389 (PMC8365765; doi:10.3389/fcell.2021.708389)
Supplement: Supplementary file 1 [file Data_Sheet_1.docx]

Supplementary Material

**Supplementary Table 1.** List of the primers used for qRT-PCR

| **Gene** | **Forward/Reverse primer (5′-3′)** | **Catalog Number** |
| --- | --- | --- |
| FOXA2 | - | HQP008906 (Genecopoeia) |
| LMX1A | - | HQP010749 (Genecopoeia) |
| TH | - | HQP018064 (Genecopoeia) |
| GIRK2 | - | HQP010010 (Genecopoeia) |
| TUJ1 | GGCCAAGGGTCACTACACG/ GCAGTCGCAGTTTTCACACTC | - |
| DAT | ACAGAGGGGAGGTGCGCCAGTTCACG/ACGGGGTGGACCTCGCTGCACAGATC | - |
| vGLUT | TCCAGGGACTCTCAGGCTAA/CATCAGAAACGCTGGTGAGA | - |
| GAD1 | TGTCCAGGAAGCACCGCCATAA/TCCTTGACGAGAATGGCAGAGC | - |
| GFAP | ATCTCCACGGTCTTCACCAC/ACATCGAGATCGCCACCTAC | - |
| PGC1-α | CCCTGCAATTGTTAAGACTGAG/TTGTTGGTTTGGCTTGTAAGTG | - |
| β-actin | TGAAGTGTGACGTGGACATC/GGAGGAGCAATGATCTTGAT | - |

**Supplementary Table 2.** Summary of passive and active electrophysiological properties of the recorded neuronal cells generated in the 2D and 3D systems.

|  | N neurons | Vm (mV) | Rm (MΩ) | Cm (pF) | Spontaneous APs / Ih | Firing freq (Hz) | Evoked AP |
| --- | --- | --- | --- | --- | --- | --- | --- |
| 2D (day 30) | 14 | -33 ± (-2) | 848 ± 127 | 13 ± 1.4 | 0% / 0% | - | 0/14 (0%) |
| 3D (day 30) | 17 | -41 ± (-2) | 705 ± 78 | 19 ± 1.9 | 35% / 41% | 2.7 ± 0.97 | 6/17 (35%) |
| 2D (day 40) | 14 | -44 ± (-3) | 489 ± 66 | 24 ± 2.6 | 36% / 43% | 10.1 ± 5.8 | 7/14 (50%) |
| 3D (day 40) | 14 | -44 ± (-1) | 352 ± 29 | 23 ± 1.6 | 36% / 71% | 7 ± 2.5 | 12/14 (86%) |
| 2D (day 50) | 10 | -49 ± (-3) | 606 ± 143 | 26 ± 3.4 | 40% / 40% | 9.1 ± 3.3 | 6/10 (60%) |
| 3D (day 50) | 12 | -47 ± (-2) | 459 ± 105 | 46 ± 5.5 | 75% / 83% | 5.7 ± 0.9 | 9/12 (75%) |

Values represent mean ± SEM, otherwise the percentage of neurons is given. Vm, resting membrane potential; Rm, input resistance; Cm, membrane capacitance; APs, action potentials; Ih, hyperpolarization-activated mixed cation current; freq, frequency.


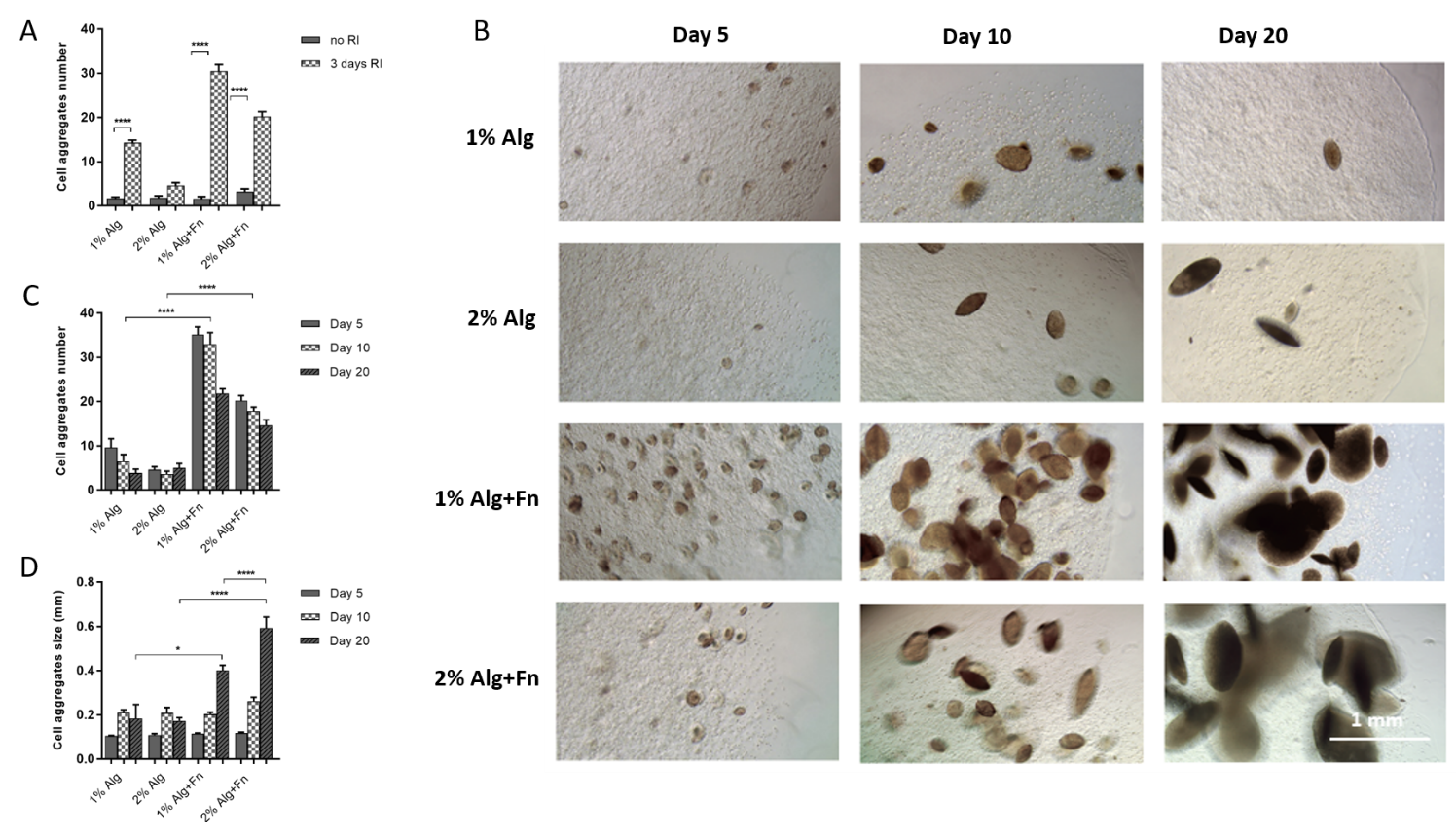


**Supplementary Figure 1. RI is beneficial for hiPSCs encapsulated in alginate beads.** **(A)** Cell aggregates of hiPSCs encapsulated in alginate beads of different compositions (Alg, alginate; Fn, fibronectin) at day 5 of neuronal differentiation, with and without pre-treatment with RI in StemMACS medium for 3 days prior to neuronal differentiation (3 days RI versus no RI); at the day of encapsulation, RI was added in both conditions. **(B)** Proliferating cells form aggregates inside the 3D matrices, which vary in abundance and size. Day 5, 10 and 20 refer to days after initiation of differentiation. **(C)** The number of aggregates within the matrices is given as cell aggregates per field of view. **(D)** Size of cell aggregates. Length of aggregates was used for non-spherical cell aggregates as indicator of size. Statistical differences were calculated by one-way ANOVA followed by Tukey's *post hoc* test to correct for multiple comparisons (B: n=3 to 11 fields of view per condition; C: n=4-128 cell aggregates per condition). Data is plotted as mean ± SEM. * p ≤ 0.05, **** p≤ 0.0001.


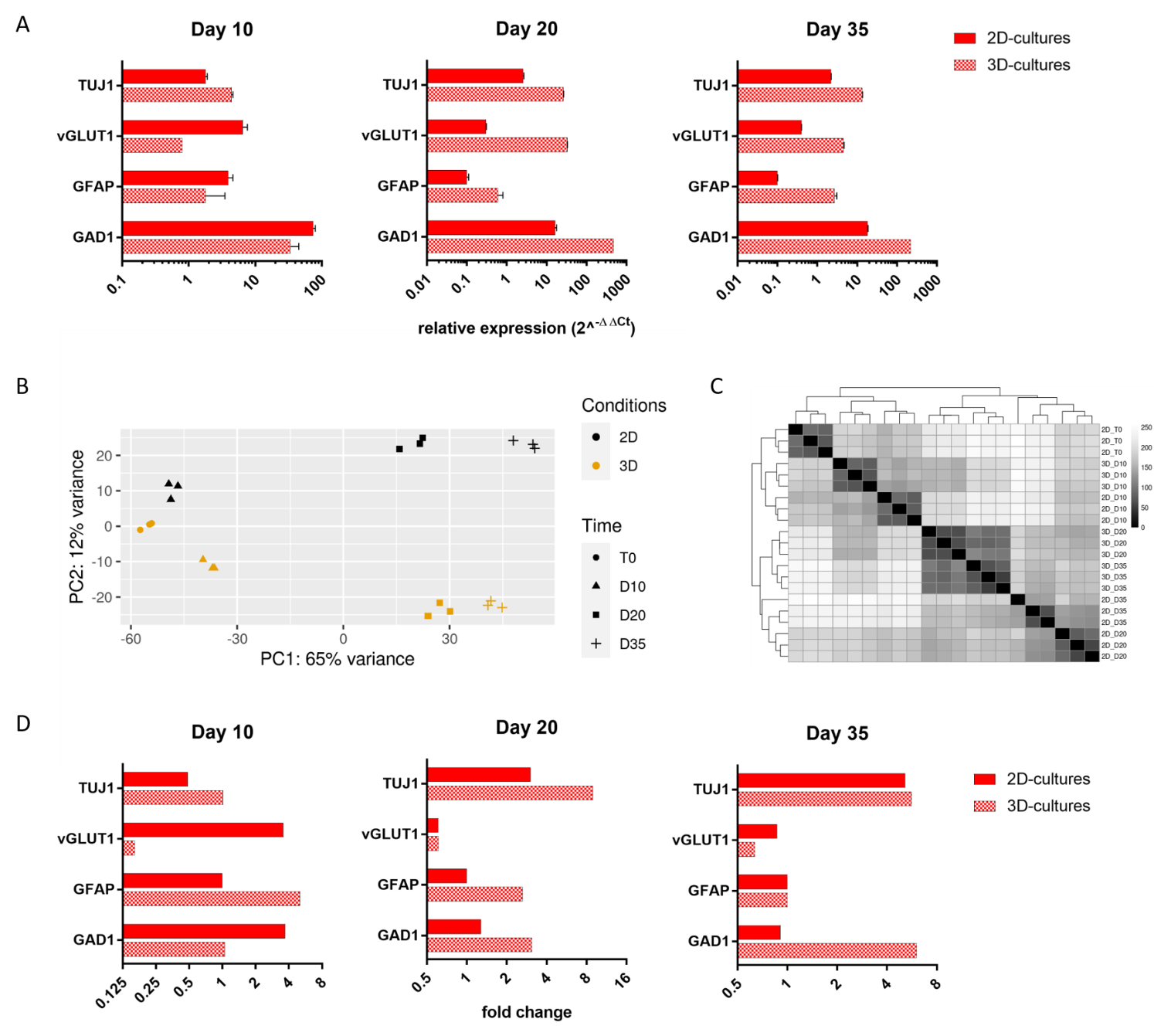


**Supplementary Figure 2. Comparative gene expression analyses of 2D and 3D cultures. (A**) qRT-PCR analysis of TUJ1, vGLUT1, GFAP, and GAD1 at days 10, 20, and 35 of differentiation. **(B)** Principal component analysis of the samples included in the RNA-seq analysis. Three replicates are represented per time point and culture condition. Different shapes encode for time points, whereas color represents culture method: black, 2D; yellow, 3D. Three samples in T0 (hiPSCs) are indicated as 3D in the plot but are used as control for both culture methods. All samples are grouped by time point and culture method.  **(C)** Heatmap depicting clustered euclidean distance between samples. Darker grey indicates samples more similar to each other. **(D)** Bars represent expression fold change at each time point (day 10, 20, 35) with respect to differentiation start for the genes TUJ1, vGLUT1, GFAP and GAD1 in the RNA-sequencing experiment. Values below 1 indicate down-regulation of expression. Red bars display gene expression change in 2D culture, whereas red and white checkered bars visualize those from 3D culture. A single fold change value is derived from three replicates for each comparison presented here.

**
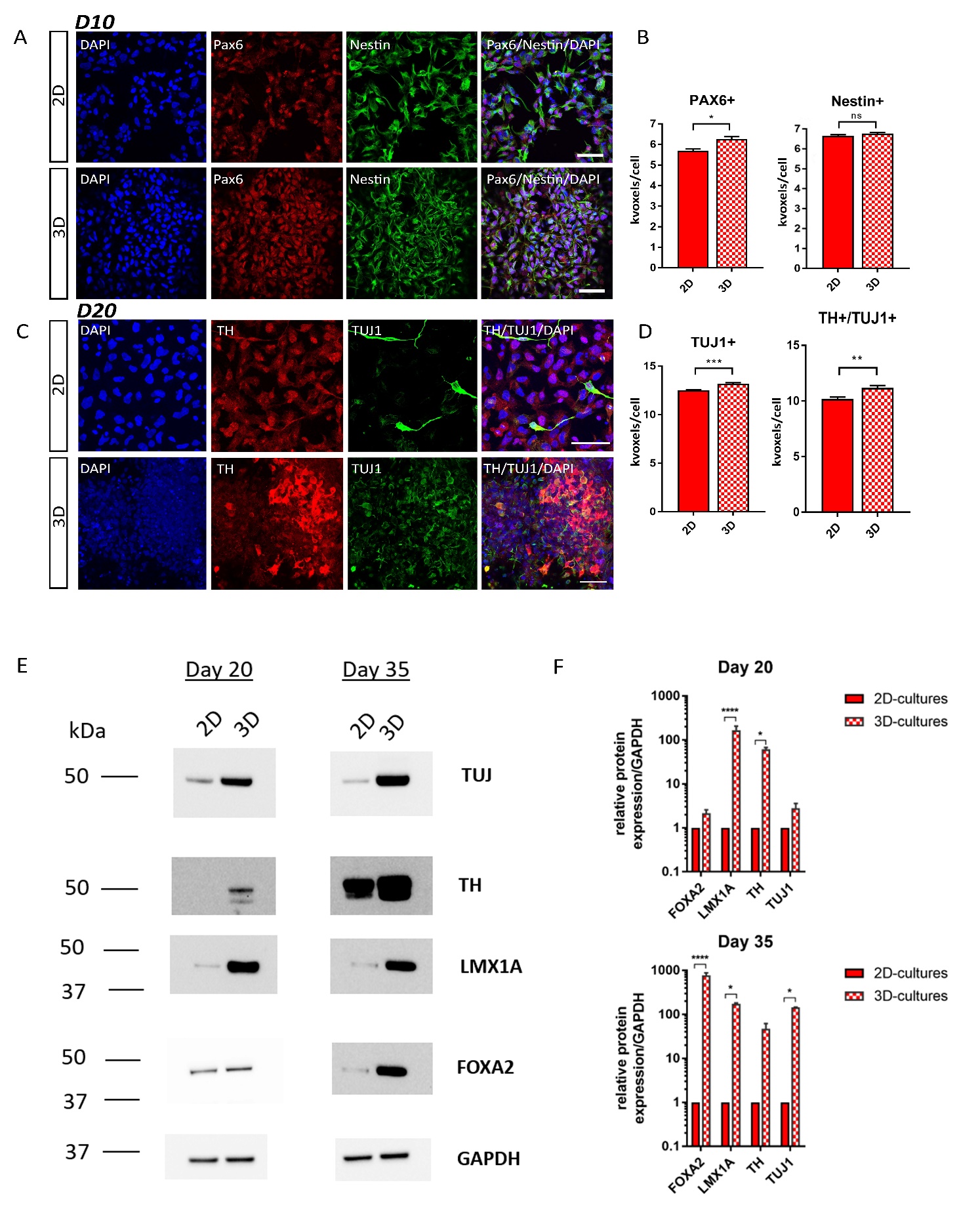
**

**Supplementary Figure 3. Comparative immunofluorescence analysis of 2D and 3D cultures.** **(A)** Representative staining of PAX6^+^ and Nestin^+^ cells at day 10 of differentiation by using positive volume (thousands of voxels, kvoxels) divided by the number of nuclei. **(B)** Quantification of PAX6^+^, Nestin^+^, and PAX6^+^/Nestin^+^ double positive cells at day 20 of differentiation. **(C)** Representative staining of TH^+^ and TUJ1^+^ cells at day 20 of differentiation. **(D)** Quantification of TH^+^, TUJ1^+^, and TH^+^/TUJ1^+^ double positive cells at day 20 of differentiation by using positive volume (thousands of voxels, kvoxels) divided by the number of nuclei. **(E)** Western blot analysis of LMX1A, FOXA2, TH, and TUJ1. **(F)** Relative density values were normalized to the loading control GAPDH. Statistical differences were calculated by two-way ANOVA followed by Sidak post hoc test to correct for multiple comparisons * p ≤ 0.05, **** p ≤ 0.0001.


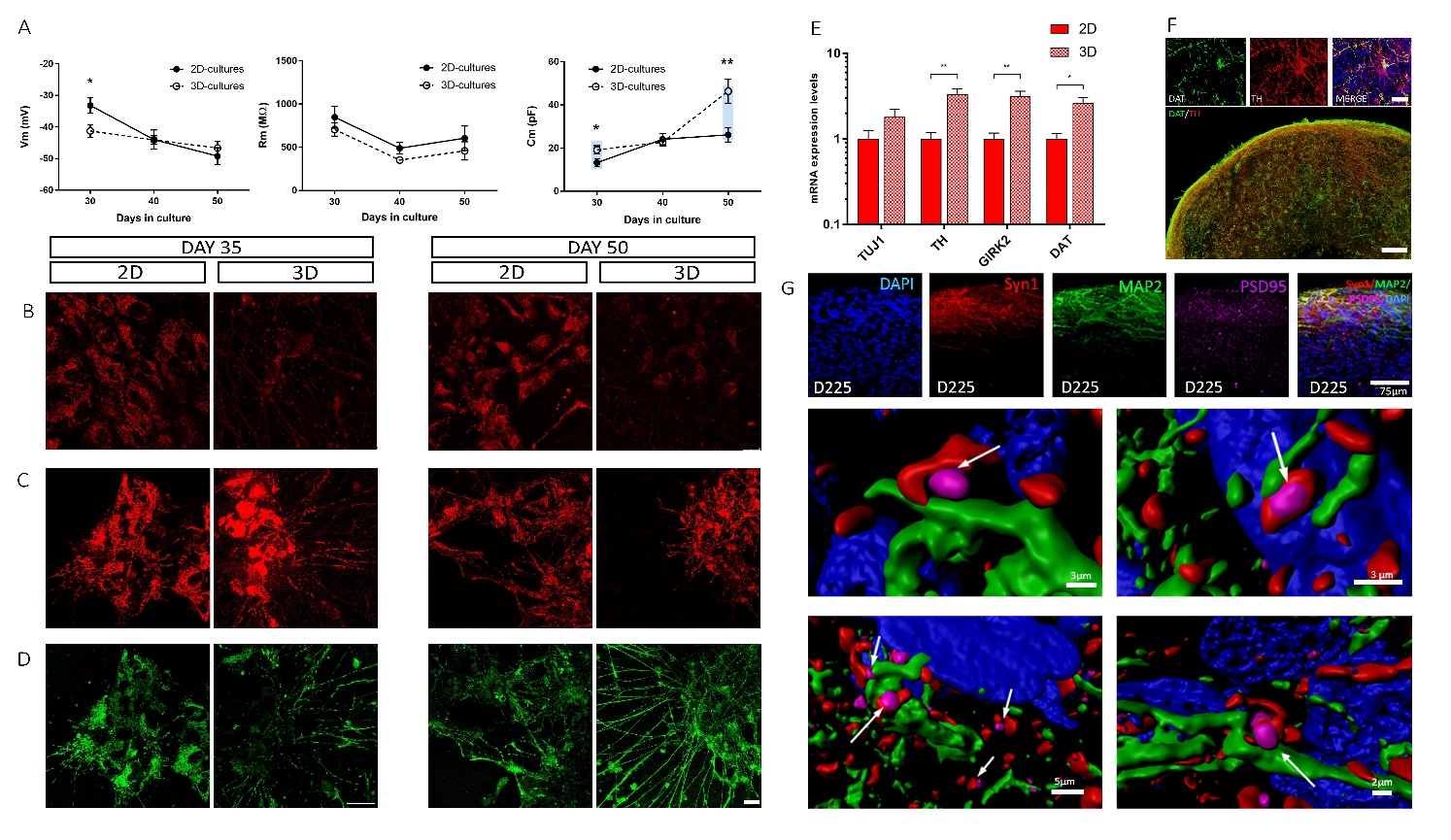


**Supplementary Figure 4.** **Electrophysiological analyses of 2D and 3D cultures. (A)** Quantification of passive electrophysiological properties of neurons in 2D and 3D cultures during neuronal differentiation at days 30, 40, and 50. APs; action potentials; Vm, resting membrane potential; Rm, input resistance; Cm, membrane capacitance. Statistical differences were calculated by multiple unpaired t-tests * p ≤ 0.05; ** p ≤ 0.01. The blue bars are illustrating an ameliorated phenotype of 3D cultures compared to the 2D counterpart. **(B)** Representative staining of mROS production, **(C)** mitochondrial membrane potential, and **(D)** mitochondrial morphology in the differentiating neurons at days 35 and 50 of differentiation. **(E)** Comparative gene expression analysis between mDA neurons differentiated in 2D and 3D culture for more than 200 days. Data are presented as the mean ± SEM from technical triplicates normalized to the gene expression in 2D culture. Statistical differences were calculated by two-way ANOVA followed by Bonferroni *post hoc* test to correct for multiple comparison * p ≤ 0.05, **p ≤ 0.01. **(F)** Immunofluorescence staining of long-term DA neural differentiation: co-staining of TH and DAT. Magnified region shows overlapping staining. Scale bar represents 100 µm. **(G)** Co-staining for the presynaptic marker protein Synapsin1 (Syn1), the neuronal marker microtubule associated protein 2 (MAP2), and the postsynaptic density protein 95 (PSD95). 3D surface reconstruction of MAP2/Syn1/PSD95 staining showing formation of synaptic puncta (white arrows).
